# The Safety of Cadonilimab: A Systematic Review and Single‐Arm Meta‐Analysis

**DOI:** 10.1002/cam4.71210

**Published:** 2025-09-03

**Authors:** Zhuo Zhang, Jiao Yu, Zhiqi Zhang, Qianxin Liu, Xiaocong Pang, Ying Zhou

**Affiliations:** ^1^ Department of Pharmacy Peking University First Hospital Beijing China; ^2^ Department of Obstetrics and Gynecology Peking University First Hospital Beijing China

**Keywords:** bispecific antibody, cadonilimab, irAEs, meta‐analysis, safety

## Abstract

**Introduction:**

Cadonilimab (AK104) is a bispecific antibody that simultaneously targets programmed cell death‐1 and cytotoxic T‐lymphocyte antigen‐4. It has received approval for the treatment of cervical cancer and gastric/gastroesophageal junction cancer. This meta‐analysis aims to assess cadonilimab's safety profile.

**Methods:**

A systematic review of electronic databases was conducted to identify clinical trials that reported cadonilimab's safety data. Immune‐related adverse events (irAEs) was the primary endpoint, and treatment‐related adverse events (TRAEs) were the secondary endpoints. A single‐group proportion meta‐analysis was conducted by R software.

**Results:**

A total of 1271 patients across more than five cancer types in 11 clinical trials were included in this study. The incidence of any grade irAEs, grade ≥ 3 irAEs, irAEs leading to treatment discontinuation, and irAEs associated with mortality was 43.3% [95% confidence interval (CI), 33.3%–53.4%], 11.3% (95% CI, 9.5%–13.3%), 3.7% (95% CI, 1.5%–6.5%), and 0% (95% CI, 0%–0.4%), respectively. Hypothyroidism was the most common all‐grade irAEs (13.3%, 95% CI, 8.9%–18.5%). The incidence of TRAEs was higher in the combined therapy group compared to the cadonilimab monotherapy group.

**Conclusions:**

The irAEs associated with cadonilimab are generally manageable. When combining with other anticancer agents, physicians and pharmacists should be particularly aware of the potential increase in TRAEs.

## 1 Introduction

Immune checkpoint inhibitors (ICIs), including targeting programmed cell death‐1 (PD‐1), programmed cell death ligand 1 (PD‐L1), and cytotoxic T‐lymphocyte antigen‐4 (CTLA‐4), have revolutionized cancer treatment, demonstrating broad applications across multiple tumor types [1]. Current clinical studies have shown that combination therapy of anti‐CTLA‐4 and anti‐PD‐(L)1 antibodies could offer a better survival benefit but have a higher incidence of all grade and grade ≥ 3 adverse events (AEs), especially immune‐related adverse events (irAEs) [2, 3]. Cadonilimab (AK104), a first‐in‐class bispecific, humanized IgG1 antibody, was designed with the goal of specifically, and ideally simultaneously, binding and inhibiting PD‐1 and CTLA‐4 on antigen‐specific T cells to overcome checkpoint inhibition [4]. Its tetravalent structure with four antigen binding sites enhances antigen binding affinity and helps to reduce off‐target effects and elicit a more efficient immune response [5]. With Fc‐null design, cadonilimab could eliminate a series of functions, like antibody‐dependent cellular cytotoxicity (ADCC), antibody‐dependent cellular phagocytosis (ADCP), complement‐dependent cytotoxicity (CDC), and cytokine release, which contribute to lower irAEs in clinical settings [6]. In June 2022, the National Medical Products Administration (NMPA) has approved cadonilimab at a dose of 6 mg/kg every 2 weeks (q2w) for the treatment of platinum‐resistant recurrent/metastatic cervical cancer (R/M CC). Additionally, in September 2024, it has received approval from NMPA for a new indication: cadonilimab at a dose of 10 mg/kg every 3 weeks (q3w) in combination with fluoropyrimidine and platinum‐based chemotherapy for the first‐line treatment of patients with locally advanced unresectable or metastatic gastric or gastroesophageal junction (G/GEJ) adenocarcinoma. Of note, the US Food and Drug Administration also granted fast track and orphan drug designations [7].

Beyond approved indications, emerging clinical data demonstrate cadonilimab's therapeutic potential across various solid cancers. A phase 1b/2 study in recurrent or metastatic nasopharyngeal carcinoma reported an objective response rate (ORR) of 26.1% with monotherapy as third‐line treatment [8]. Furthermore, combination strategies with targeted agents (e.g., lenvatinib) or chemotherapy (e.g., paclitaxel + cisplatin/carboplatin) also show enhanced efficacy in advanced hepatocellular carcinoma [9] and R/M CC [10]. Despite generally manageable toxicity profiles of cadonilimab, critical knowledge gaps persist regarding toxicity variations across dosing regimens (6 mg/kg q2w vs. 10 mg/kg q3w), combination therapies, and cancer types. Limited data exist on AE‐related discontinuation rates and mortality rates among patients receiving cadonilimab. Based on these premises, we carried out a systematic review and single‐arm meta‐analysis to comprehensively evaluate the incidence and spectrum of irAEs and treatment‐related adverse events (TRAEs) with cadonilimab in clinical trials.

## 2 Materials and Methods

A search for peer‐reviewed articles on cadonilimab was performed in PubMed, Embase, the Cochrane Library, and ClinicalTrials.gov. The search encompassed all records published or registered from database inception up to February 20, 2025. The complete search string used across all databases was: (“cadonilimab” OR “AK104”). Study types were restricted to clinical trials. Only studies published in the English language were considered. This systematic review and meta‐analysis was registered with the International Prospective Register of Systematic Reviews (PROSPERO) numbered with CRD420250654976. The literature was screened with EndNote X9. Two authors independently conducted the literature search and study selection, and any inconsistencies were resolved via a group discussion until a consensus was reached.

### 2.1 Inclusion and Exclusion Criteria

Inclusion criteria included clinical trials that met the following criteria: (1) the patients received treatment with cadonilimab, either alone or in combination with other anticancer drugs; (2) the study type was a clinical trial; (3) the study subjects were patients with malignant tumors; and (4) adverse events (AEs) or safety data were reported.

Exclusion criteria included the following: (1) types of studies other than clinical trials; (2) conference reports; (3) not a full text; and (4) reanalysis of clinical trials.

### 2.2 Outcome Indicators

irAEs was the primary outcome. And treatment‐related adverse events (TRAEs) were the secondary outcome. AEs were graded with the use of the National Cancer Institute Common Terminology Criteria for Adverse Events (NCI‐CTCAE) and coded according to the Medical Dictionary for Regulatory Activities.

### 2.3 Data Extraction and Literature Quality Evaluation

A comprehensive data extraction of information on the specific study (study ID, author, country, sample size, and inclusion and exclusion criteria), therapeutic regimen (medicine, dose, frequency, and duration), general information about the patients (age, and sex), and outcomes (type and severity of AEs) was performed for all the included literature with a specially designed form. All types of AEs of different grades and the number of participants were extracted. The rates of total AEs and grade 3 or higher (grade ≥ 3) AEs were evaluated separately. We used the Cochrane Handbook for Systematic Reviews of Interventions (version 6.5) [11] to evaluate the bias risk of randomized controlled trials, and the Methodological Index for Non‐Randomized Studies (MINORS) tool was used to assess the quality of the remaining included studies [12].

### 2.4 Statistical Analysis

Single‐group proportion meta‐analyses were conducted using the meta package (8.0‐2) in R (4.41). Pooled estimates with 95% confidence intervals (CIs) were calculated. Proportions were transformed via the Freeman‐Tukey double arcsine method. Heterogeneity across studies was quantified using the Paule‐Mandel estimator, and a random‐effects model was used in the presence of substantial heterogeneity (*I* ^2^ > 50%). Sensitivity analyses were conducted to evaluate the robustness and reliability of the combined results. Funnel plot and Egger's tests were conducted to assess for publication bias with respect to the investigated outcomes, which include more than 10 studies [13].

### 2.5 Subgroup Analyses

Subgroup analyses were conducted for irAEs to evaluate specific treatments, cancer types, and dosage regimens among elements suspected of influencing outcome variability. Based on differing treatment strategies, the included studies were categorized into the following three subgroups: cadonilimab monotherapy group (group A), cadonilimab combined with chemotherapy ± targeted therapy group (group B), and cadonilimab combined with targeted therapy group (group C).

## 3 Results

### 3.1 The Literature Search

**FIGURE 1:**
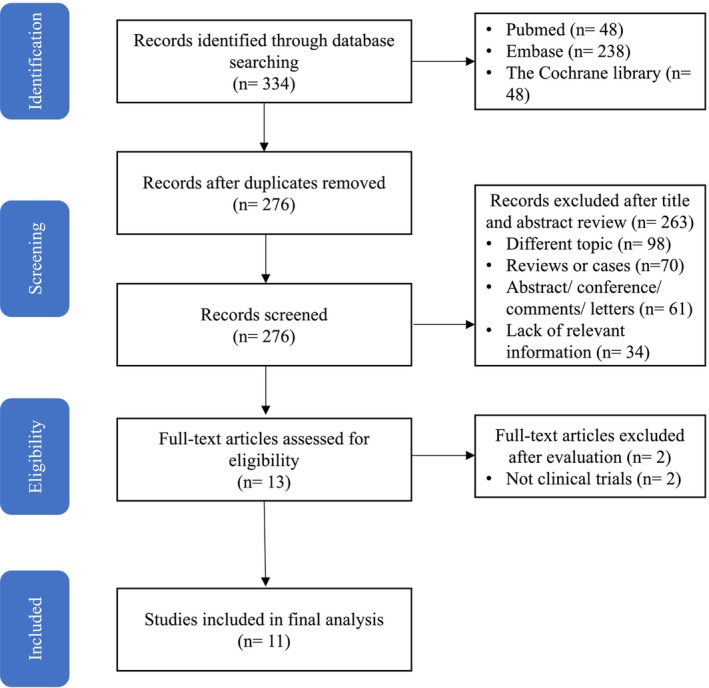
Flow diagram of the literature search and studies selection process.

The diagram of the study selection process is presented in Figure 1. Overall, 334 citations were identified by the initial search, and 11 different articles (1271 patients) across more than five cancer types were included in the final analysis. A manual search of the reference lists of these studies did not yield any new eligible studies.

### 3.2 Characteristics and Quality of the Studies

The general characteristics of the included studies are presented in Table 1. These studies were published from 2023 to 2025. Ten of these were conducted in China and one in Australia. Of the 11 included studies [8, 9, 10, 14, 15, 16, 17, 18, 19, 20, 21], one was a phase 1 trial, six were phase 1b/2 trials, two were phase 2 trials, and two were phase 3 trials. Regarding cancer types, three studies were conducted in patients with G/GEJ adenocarcinoma, two in patients with R/M CC, two in patients with non‐small cell lung cancer (NSCLC), one in patients with nasopharyngeal carcinoma, one in patients with hepatocellular carcinoma, and two in patients with multiple solid tumors. Seven studies' treatments were cadonilimab combined with chemotherapy or targeted therapies, and four studies' treatments were only cadonilimab with different dosages. Other information, including study sample size, treatment regimens (line, dose, frequency, and duration), patients' general information (age, and sex), and versions of NCI‐CTCAE was also listed in Table 1.

**TABLE 1:** Baseline characteristics of the studies included in the systematic review and meta‐analysis.

Author	Year	Trial No.	Coutry	Study type	Cancer type	Sample size^a^ (*n*)	Treatment line	Intervention	Median treatment duration (range; months)	Median age (range; years)	Female, *n* (%)	Versions of NCI CTCAE
Shen, L.	2025	NCT05008783	China	A multicenter, randomized, double‐blind, placebo‐controlled phase 3 study	Advanced G/GEJ adenocarcinoma	305	1	Cadonilimab 10 mg/kg q3w + XELOX q3w	5.62 (0.5–22.2)	64 (29–75)	66 (21.6%)	5
Wu, X.	2024	NCT04982237	China	A multicenter, randomized, double‐blind, placebo‐controlled phase 3 study	Persistent, recurrent, or metastatic cervical cancer	226	1	Cadonilimab 10 mg/kg q3w + cisplatin/carboplatin‐paclitaxel ± bevacizumab q3w	10.4 (IQR 4.2–22.5)	56 (23–75)	222 (100%)	5
Lou, H.	2024	NCT04868708	China	A multicenter, open‐label, phase 2 trial	Recurrent and/or metastatic cervical cancer	45	1	Cadonilimab 15/10 mg/kg q3w + cisplatin/carboplatin‐paclitaxel ± bevacizumab q3w	7–7.2	52.9 (33–71)	45 (100%)	5
Long, B.	2024	ChiCTR2200066893	China	A multicenter, phase 2 study	HER2 negative, locally advanced G/GEJ adenocarcinoma	38	1	Cadonilimab 10 mg/kg q3w + FLOT q3w	Not reported	57.0 (38–74)	4 (10.5%)	5
Gao, X.	2024	CTR20182027	China	A multicenter, open‐label, phase 1b/2 study	HER2 negative, unresectable advanced or metastatic G/GEJ adenocarcinoma	94	1	Cadonilimab 4/6/10 mg/kg q2w or 15 mg/kg q3w + (m)XELOX q3w	6.5	62.7 (29–75)	28 (29.8%)	5
Chen, Q.	2024	NCT04220307	China	A multicenter, open‐label, phase 1b/2 study	Recurrent or metastasis nasopharyngeal carcinoma	23	3	Cadonilimab 6 mg/kg q2w	Not reported	43.8 (mean)	3 (13.0%)	5
Chen, B.	2024	NCT04646330	China	A multicenter, open‐label, phase 1b/2 study	Advanced NSCLC	69	1	Cadonilimab 15/10 mg/kg q3w + anlotinib	Not reported	63.5 (40–74)	11 (15.9%)	5
Zhao. Y.	2023	NCT04172454	China	A multicenter, open‐label, phase 1b/2 study	Advanced NSCLC	53	≥ 2	Cadonilimab 6 mg/kg q2w	4	59.0 (43–71)	10 (18.9%)	5
Qiao, Q.	2023	NCT04444167	China	A multicenter, open‐label, phase 1b/2 study	Advanced hepatocellular carcinoma	59	1	Cadonilimab 6 mg/kg q2w or 15 mg/kg q3w + lenvatinib	Not reported	56.3 (24–72)	11 (18.6%)	5
Gao, X.	2023	NCT03852251	China	A multicenter, open‐label, phase 1b/2 study	Unresectable advanced solid tumors	240	≥ 2	Cadonilimab 6/10 mg/kg or 450 mg q2w	Not reported	Phase 1b: 56 (51–63); phase 2: 51–63	165 (68.7%)	5
Frentzas, S.	2023	NCT03261011	Australia	A multicenter, phase 1 study	Advanced or metastatic solid tumor	119	≥ 1	Cadonilimab 0.2–2/4/6/10 mg/kg q2w or 450 mg q2w or 15/25 mg/kg q3w	3.0 (0.5–26.6)	61.0 (20–85)	52 (43.7%)	4.03

Abbreviations: FLOT, docetaxel, oxaliplatin, leucovorin, and 5‐fluorouracil; G/GEJ, gastric or gastroesophageal junction; HER2, human epidermal growth factor receptor 2; IQR, interquartile range; NCI‐CTCAE, National Cancer Institute's Common Terminology Criteria for Adverse Events; NSCLC, non‐small cell lung cancer; XELOX, oxaliplatin and capecitabine.

^a^ Included in cadonilimab group for safety analysis.

The results of the evaluation of the quality of the studies are shown in Tables S1 and S2. Of two randomized controlled trials (RCTs), one study was rated as having some concerns regarding the overall risk of bias, and the other had a low risk of bias. Among the nine included non‐comparative studies evaluated with the MINORS scale, one study scored 15 points, while the remaining eight studies achieved scores ≥ 16 points. The majority of studies demonstrated relatively high methodological quality.

### 3.3 irAE Rates

**FIGURE 2:**
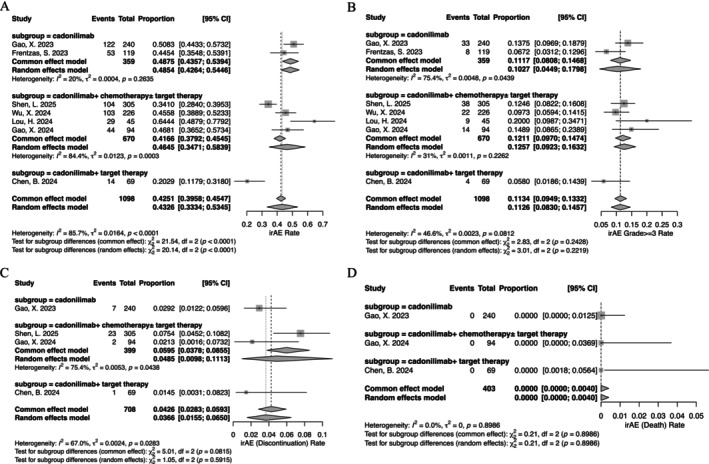
Incidence of immune‐related adverse events (irAEs). (A) All‐grade irAEs; (B) grade ≥ 3 irAEs; (C) irAEs leading to treatment discontinuation; (D) irAEs associated with mortality.

The occurrence of any irAEs was 43.3% (95% CI, 33.3%–53.4%; *I* ^2^, 85.7%), and the rates of grade ≥ 3 irAEs were 11.3% (95% CI, 9.5%–13.3%; *I* ^2^, 46.6%). The frequencies of irAEs leading to treatment discontinuation were 3.7% (95% CI, 1.5%–6.5%; *I* ^2^, 67.0%). Lastly, the proportions of irAEs associated with mortality were 0% (95% CI, 0%–0.4%; *I* ^2^, 0%) (Figure 2).

A total of 68 distinct types of irAEs were reported across 11 studies (Table S3), but only six irAEs were documented by at least three studies (Table 2). The most prevalent all‐grade irAE was hypothyroidism, with an incidence of 13.3% (95% CI, 8.9%–18.5%; *I* ^2^, 83.7%). Hyperthyroidism (9.4%; 95% CI, 6.6%–12.6%; *I* ^2^, 72.4%) and hyperglycemia (2.7%; 95% CI, 1.7%–4.0%; *I* ^2^, 43%) were also commonly reported as immune‐related endocrine AEs. Additionally, immune‐related cutaneous AEs were equally frequent, with rash and pruritus occurring in 7.6% (95% CI, 2.8%–14.2%; *I* ^2^, 89.6%) and 3.7% (95% CI, 0.2%–10.2%; *I* ^2^, 88.2%), respectively.

**TABLE 2:** Meta‐analysis of the incidence of irAEs.

irAEs	All‐grade irAEs			Grade ≥ 3 irAEs		
	Studies, *n*	Proportions (95% CI)	*I* ^2^	Studies, n	Proportions (95% CI)	*I* ^2^
Hypothyroidism	8	13.3% (8.9%–18.5%)	83.7%	7	0% (0%–0.3%)	0%
Hyperthyroidism	7	9.4% (6.6%–12.6%)	72.4%	6	0% (0%–0.4%)	0%
Rash	7	7.6% (2.8%–14.2%)	89.6%	6	0.5% (0.1%–1.3%)	33.5%
Pruritus	4	3.7% (0.2%–10.2%)	88.2%	4	0% (0%–0.3%)	0%
Hyperglycaemia	3	2.7% (1.7%–4.0%)	43.0%	2	0.9% (0.2%–1.9%)	0%
Immune‐mediated hepatitis	3	1.3% (0.3%–2.6%)	0%	3	1.3% (0.3%–2.6%)	0%

Abbreviation: irAEs, immune‐related adverse events.

A subgroup analysis stratified by distinct treatment modalities was conducted, although the limited number of trials within each subgroup precludes definitive conclusions. The results indicated a significant difference in the incidence of any irAEs across the three treatment groups (*p* < 0.0001). However, no statistically significant differences were observed among other outcomes (Figure 2). In the subgroup analysis of tumor types, owing to the limited number of studies involving certain tumor types, only CC and G/GEJ were specifically distinguished, while the remaining types were categorized collectively as other solid tumors. The findings indicated that the incidence of irAEs in CC was numerically higher, at 53.5% (95% CI, 43.1%–63.7%; *I* ^2^, 69.4%). The occurrence of any irAEs in G/GEJ was 39.6% (95% CI, 27.7%–52.3%; *I* ^2^, 79.4%). However, no significant difference was observed in the overall incidence of irAEs and other outcomes across the different tumor types analyzed (Figure S1). The safety profile was also compared between the dosing groups. For the two dosages currently approved by the NMPA, 6 mg/kg q2w and 10 mg/kg q3w, the incidences of irAEs were 50.3% (95% CI, 44.3%–56.4%; *I* ^2^, 24.1%) and 41.9% (95% CI, 28.2%–56.4%; *I* ^2^, 74.4%), respectively. The incidences of grade ≥ 3 were 11.2% (95% CI, 4.8%–19.5%; *I* ^2^, 64.8%) and 10.4% (95% CI, 7.8%–13.2%; *I* ^2^, 0%), respectively. The results of other dosing regimens are presented in Figure S2.

### 3.4 TRAE Rates

**FIGURE 3:**
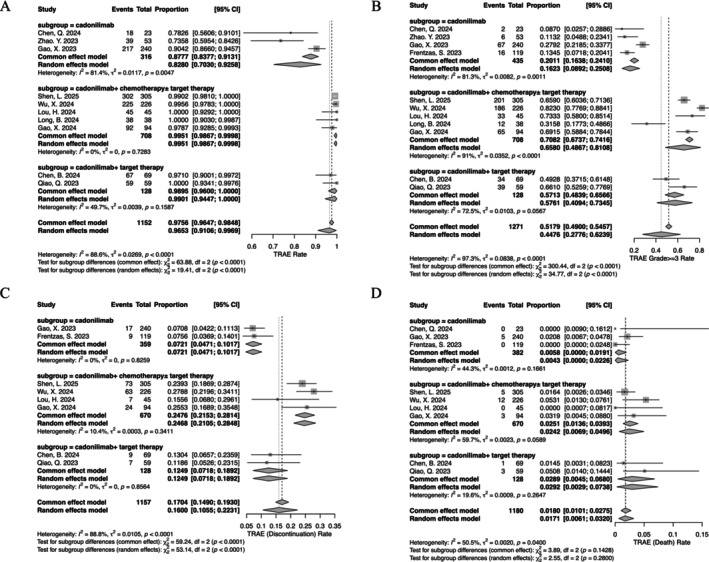
Incidence of treatment‐related adverse events (TRAEs). (A) All‐grade TRAEs; (B) grade ≥ 3 TRAEs; (C) TRAEs leading to treatment discontinuation; (D) TRAEs associated with mortality.

Given the significant influence of various treatment regimens on TRAEs, this section emphasizes the reporting of subgroup analysis results. The incidences of any TRAEs in groups A, B, and C were 82.8% (95% CI, 70.3%–92.6%; *I* ^2^, 81.4%), 99.5% (95% CI, 98.7%–100%; *I* ^2^, 0%), and 98.9% (95% CI, 96.0%–100%; *I* ^2^, 49.7%), respectively. The rates of grade ≥ 3 TRAEs in groups A, B, and C were 16.2% (95% CI, 8.9%–25.1%; *I* ^2^, 81.3%), 65.8% (95% CI, 48.7%–81.1%; *I* ^2^, 91%), and 57.6% (95% CI, 40.9%–73.4%; *I* ^2^, 72.5%), respectively. The frequencies of TRAEs leading to treatment discontinuation in groups A, B, and C were 7.2% (95% CI, 4.7%–10.2%; *I* ^2^, 0%), 24.8% (95% CI, 21.5%–28.1%; *I* ^2^, 10.4%), and 12.5% (95% CI, 7.2%–18.9%; *I* ^2^, 0%), respectively. Lastly, the proportions of TRAEs associated with mortality in groups A, B, and C were 0.6% (95% CI, 0%–1.9%; *I* ^2^, 44.3%), 2.4% (95% CI, 0.7%–5.0%; *I* ^2^, 59.7%), and 2.9% (95% CI, 0.4%–6.8%; *I* ^2^, 19.6%), respectively (Figure 3).

### 3.5 Sensitivity Analysis and Publication Bias

The conclusions remained robust and unchanged after sequentially excluding each study from the overall analyses of all‐grade irAEs, grade ≥ 3 irAEs, and irAEs leading to discontinuation or death (Figure S3). With regard to the six specific irAEs in Table 2, the results of the sensitivity analyses found that the conclusions were not altered by sequentially excluding an individual study (Figure S4). Given that the number of studies included in each outcome did not exceed 10, publication bias assessments were not conducted.

## 4 Discussion

To our knowledge, this is the first systematic review and meta‐analysis assessing the safety profile of cadonilimab in patients with advanced cancer. Based on data from 11 clinical trials involving 1271 patients, the incidence of any grade irAEs was 43.3%. Hypothyroidism was the most common all‐grade irAEs, occurring in 13.3% of patients, and no new safety signals were observed. The incidence of grade ≥ 3 irAEs, irAEs leading to treatment discontinuation, and irAEs associated with mortality was 11.3%, 3.7%, and 0%, respectively. These incidence rates may be helpful for oncologists when deciding between ICI regimens and in planning future studies using cadonilimab.

In comparison with ICIs monotherapy, our findings indicate that the incidence of any grade and grade ≥ 3 irAEs of cadonilimab was higher than those of PD‐1 inhibitors (26.5%; 7.1%) or PD‐L1 inhibitors (17.1%; 6.3%) as monotherapy, yet lower than those of CTLA‐4 inhibitors (53.8%; 21.5%) as monotherapy [22]. Regarding ICIs combination therapy, the rates of all‐grade irAEs of cadonilimab were comparable to those of ICIs combination regimens (45.7%) [23], and even lower than the rate observed with PD‐L1 + CTLA‐4 inhibitors (61.1%) [22]. Notably, the occurrence of grade ≥ 3 irAEs of cadonilimab was lower than PD‐1 + CTLA‐4 (54.8%) and PD‐L1 + CTLA‐4 (16.7%) combination therapies. This observed safety advantage may be mechanistically explained by cadonilimab's unique bispecific design. Its tetravalent structure enhances target specificity and immune synapse formation, potentially reducing off‐target effects [5]. Critically, cadonilimab incorporates an IgG1 scaffold with Fc engineering designed to eliminate binding to FcγRs (including FcγRIa, FcγRIIa, FcγRIIIa variants) and C1q [24]. As demonstrated in cellular assays, this Fc‐null design abolishes FcγR‐mediated effector functions, such as ADCC, CDC, or ADCP [6, 24]. Furthermore, it induces only minimal secretion of pro‐inflammatory cytokines IL‐6 and IL‐8 by human macrophages [24]. These intrinsic properties of cadonilimab likely avoid nonspecific interactions with immune effector cells, thereby reducing the risk of triggering irAEs. Given the critical role of dual immunotherapy in overcoming immune resistance and enhancing treatment efficacy, it has emerged as a promising direction for future advancements in immunotherapy [25]. However, prior studies have indicated that ICIs combination may increase the likelihood of adverse reactions compared to monotherapy [22, 25, 26]. Our data indicate that the incidence of irAEs with cadonilimab lies between that of PD‐(L)1 and CTLA‐4 inhibitors, and is favorable when compared to conventional ICI two‐drug combination therapy. Additionally, we anticipate future studies that will further compare the efficacy and safety profiles of cadonilimab with those of combination ICIs therapies.

Both cadonilimab monotherapy and its combination with chemotherapy have demonstrated promising antitumor efficacy in solid tumors [8, 15, 16]. The Compassion‐15 study evaluated the use of cadonilimab in combination with chemotherapy as a first‐line treatment for HER2‐negative advanced G/GEJ cancer. The results indicated that cadonilimab combined with chemotherapy significantly improved overall survival (hazard ratio (HR) 0.66; 95% CI 0.54–0.81; *p* < 0.001) [14]. The Compassion‐16 study revealed that cadonilimab in combination with traditional taxane + platinum chemotherapy ± bevacizumab, administered as first‐line therapy for R/M CC, significantly enhanced both progression‐free survival (HR 0.62; 95% CI 0.49–0.80; *p* < 0.0001) and overall survival (HR 0.64; 95% CI 0.48–0.86; *p* = 0.0011). In our further subgroup analysis of irAEs, we observed that the incidence of irAEs was comparable between cadonilimab monotherapy and cadonilimab combined with chemotherapy ± targeted therapy (Figure 2). This exploratory analysis suggests that the addition of chemotherapy ± targeted therapy may not substantially increase the risk of irAEs. Our study also incorporated two investigations evaluating cadonilimab in combination with oral targeted therapy [9, 18], but only one of these studies reported data related to irAEs [18], indicating a numerically lower overall irAE incidence compared to other treatment groups. However, given the limited number of studies included, these conclusions are preliminary, and further research is warranted to validate these observations. In the analysis of TRAEs, subgroup analyses indicated that the overall incidence of TRAEs, grade ≥ 3 TRAEs, TRAEs leading to treatment discontinuation, and mortality were higher in the combined therapy group compared to the monotherapy group, particularly in the combined chemotherapy group. Although these differences reached nominal statistical significance, they should be interpreted with caution and regarded as hypothesis‐generating only. This finding underscores the necessity to comprehensively evaluate the safety profiles of other anti‐cancer agents included in the regimen, and the differentiation of non‐irAEs and drug–drug interactions is more challenging with cadonilimab combination treatments.

Tumors exhibit marked heterogeneity, leading to significant variability in their response to ICIs. “Hot” tumors may demonstrate greater efficacy from immunotherapy but are also associated with an increased risk of irAEs [27]. For instance, irrespective of whether PD‐(L)1 monotherapy or combination ICI regimens were used, melanoma (37.5%) and gastric cancer (12.5%) emerged as primary tumors with higher incidence rates of immune‐related cardiovascular events. Gynecological cancers exhibited an incidence rate of 7.5%, while lung cancer and lymphoma showed a lower incidence at 5% [28]. Our results noted that the incidence of irAEs in CC might be higher compared to G/GEJ cancers and other solid tumors, although no statistically significant differences were observed between the groups. However, this comparison was based on only two to three studies per subgroup, resulting in low statistical power and substantial uncertainty. Additionally, due to the limited number of included studies, subgroup analyses for other “hot” tumors, such as NSCLC, could not be conducted. It is important to note that conflicting results remain regarding the correlation between the incidence of irAEs and cancer types. A large meta‐analysis encompassing over 20,000 patients from 125 clinical trials indicated that the incidence of irAEs was consistent across all cancer types [29]. This may be attributable to variations in the definition, assessment criteria, follow‐up duration, diagnostic methodologies, and reporting standards for irAEs across different studies [27]. Future high‐quality research is anticipated to provide a more precise evaluation of the relationship between the incidence of cadonilimab‐associated irAEs and various tumor types.

The present analysis exclusively incorporated clinical trials, which inherently exhibited considerable variation in dosing regimens. To address this heterogeneity, our study stratified the administered doses into up to eight distinct subgroups, including the two NMPA‐approved regimens—6 mg q2w and 10 mg q3w. Subgroup analyses revealed that, although doses were aggregated across comparable regimens, substantial heterogeneity persisted within certain subgroups. For instance, in the analysis of all‐grade irAEs, the 10 mg q3w subgroup demonstrated a heterogeneity of 74.4%, which may be attributed to the limited number of included studies (ranging from 1 to 6 studies per subgroup) and variability in tumor histologies across the trials. Our findings indicated no statistically significant differences in the overall incidence of irAEs or the incidence of grade ≥ 3 irAEs among the different dosing groups (Figure S2). Nevertheless, further large‐scale, real‐world studies are warranted to more precisely evaluate the safety profiles associated with specific dosing regimens.

The present meta‐analysis has also some limitations. First, a key limitation is the exclusive inclusion of RCTs, which excluded real‐world evidence. Although this approach ensured a high level of methodological rigor and homogeneity of safety data for the meta‐analysis, it inherently restricts the ability to detect rare irAEs and evaluate long‐term or delayed‐onset toxicities beyond typical trial follow‐up. Furthermore, the strict eligibility criteria of RCTs limit the generalizability of findings to more complex patient populations encountered in real‐world clinical practice. Consequently, our findings may underestimate cadonilimab's rare and long‐term irAE risks. A comprehensive assessment of its safety profile will therefore require complementary evidence from prospective observational studies and robust pharmacovigilance systems. Second, there may be incomplete information in the included studies. For instance, four studies reported TRAEs but omitted data on irAEs [8, 9, 16, 19]. Furthermore, each study typically only documented common AEs, with varying cut‐off values for reporting the incidence of specific irAEs or TRAEs (e.g., 10%, 20%). Third, as previously discussed, the definitions and assessment criteria for irAEs may not be standardized across studies, potentially leading to an underestimation of the true incidence of cadonilimab‐associated irAEs. Fourth, there is considerable heterogeneity among the studies, which has been mitigated through sensitivity analysis and subgroup analysis in this review. The results of the sensitivity analysis indicate stable conclusions. However, in the subgroup analysis, some subgroups include a limited number of studies, which may compromise statistical power. Larger‐scale studies could be conducted focusing on smaller sample subgroups, such as those involving combination oral target therapy, to elucidate the risk profiles associated with different treatments, dosing regimens, or specific tumor types. Fifth, the pooled safety data primarily represent outcomes in Chinese patients, who constituted 90.6% of the study population, with limited representation from other ethnic groups. Prospective validation in multi‐regional cohorts, particularly those encompassing non‐Asian populations, is necessary to confirm the generalizability of these findings. Sixth, while this study did not perform a publication bias test due to the limited number of included studies, it is important to note that this meta‐analysis may still be susceptible to publication bias. Seventh, a key limitation of single‐arm meta‐analyses is the absence of a control group, which precludes direct attribution of differences in safety outcomes. Future research could address this by conducting RCTs to directly compare the safety profiles of cadonilimab versus PD‐1 + CTLA‐4 combination therapy.

## 5 Conclusions

This analysis aims to provide a deeper understanding of cadonilimab's safety profile and inform clinical decision‐making in diverse oncological settings. In summary, the irAEs associated with cadonilimab are generally manageable. However, when combining it with other anti‐tumor agents, it is crucial to be vigilant regarding the potential increase in TRAEs. A comprehensive assessment of the treatment regimen's safety profile should be conducted, and patients should be provided with thorough pharmaceutical care to reduce risks and ensure optimal outcomes.

## Author Contributions

**Zhuo Zhang:** conceptualization, data curation, formal analysis, methodology, software, visualization, and writing – original draft preparation. **Jiao Yu:** data curation, formal analysis, and writing – original draft preparation. **Zhiqi Zhang:** validation, software, and visualization. **Qianxin Liu:** supervision, and writing – review and editing. **Xiaocong Pang:** conceptualization, methodology, writing – review and editing, and funding acquisition. **Ying Zhou:** project administration, supervision, and writing – review and editing.

## Conflicts of Interest

The authors have declared no conflicts of interest.

## Supporting information


**Figure S1:** Incidence of immune‐related adverse events (irAEs) organized by cancer types.

**Figure S2:** Incidence of immune‐related adverse events (irAEs) organized by dosing groups.

**Figure S3:** Sensitive analysis of immune‐related adverse events (irAEs).

**Figure S4:** Sensitive analysis of specific immune‐related adverse events (irAEs).

**Table S1:** Risk of bias in randomized trials.

**Table S2:** The revised and validated version of MINORS.

**Table S3:** Summary of irAEs.

## Data Availability

The data that support the findings of this study are available in the Supporting Information of this article.
