# Supplementary material for: The Safety of Cadonilimab: A Systematic Review and Single‐Arm Meta‐Analysis
Source: Cancer Med. 2025 Sep 3;14(17):e71210. doi: 10.1002/cam4.71210 (PMC12405967; doi:10.1002/cam4.71210)
Supplement: Supplementary file 1 — Figure S1: Incidence of immune‐related adverse events (irAEs) organized by cancer types. [file CAM4-14-e71210-s005.pdf]

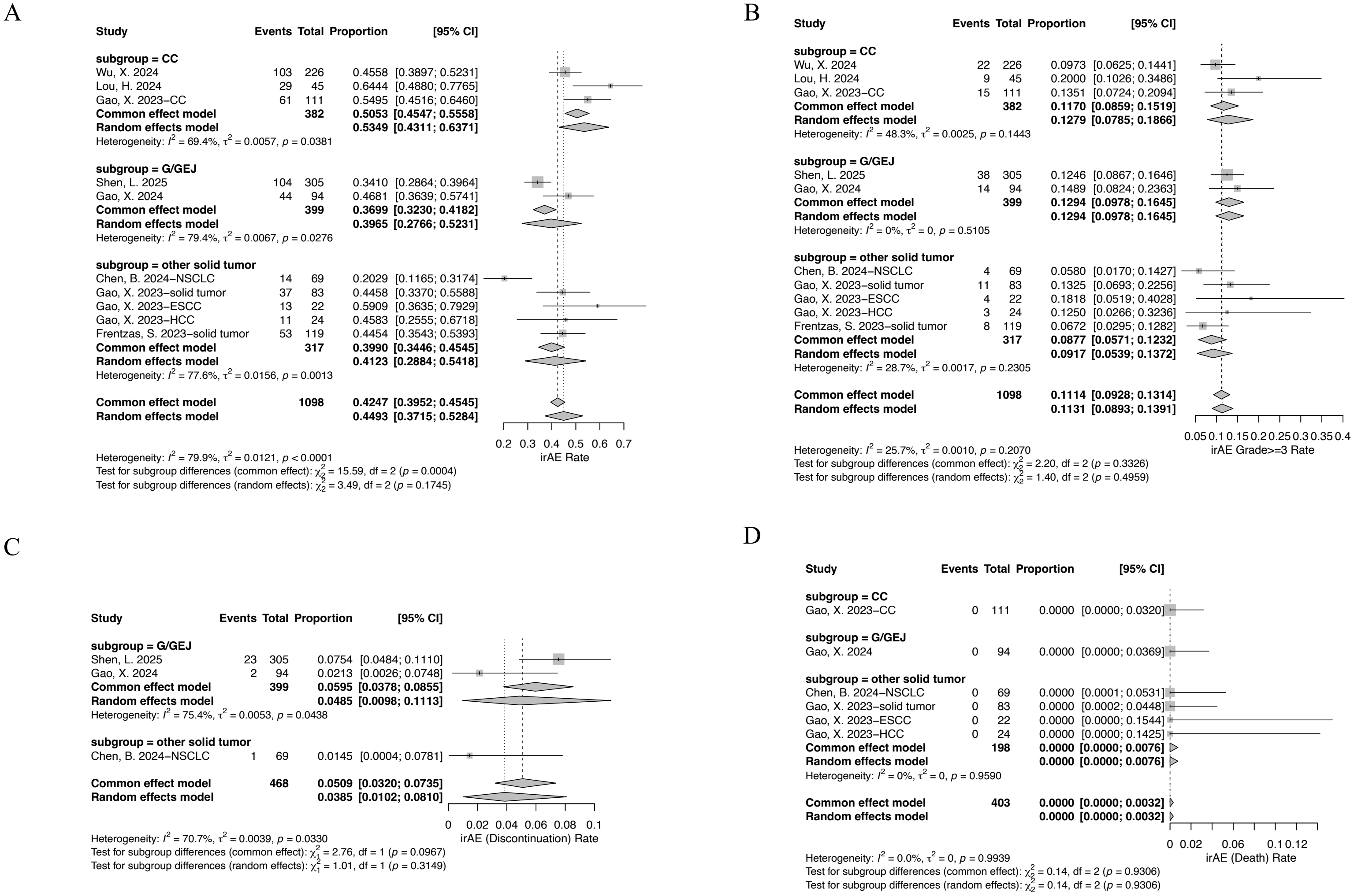
A
Study Events Total Proportion [95% CI]
subgroup = CC
Wu, X. 2024 103 226 0.4558 [0.3897; 0.5231]
Lou, H. 2024 29 45 0.6444 [0.4880; 0.7765]
Gao, X. 2023−CC 61 111 0.5495 [0.4516; 0.6460]
Common effect model 382 0.5053 [0.4547; 0.5558]
Random effects model 0.5349 [0.4311; 0.6371]
Heterogeneity: I^2 = 69.4%, τ^2 = 0.0057, p = 0.0381
subgroup = G/GEJ
Shen, L. 2025 104 305 0.3410 [0.2864; 0.3964]
Gao, X. 2024 44 94 0.4681 [0.3639; 0.5741]
Common effect model 399 0.3699 [0.3230; 0.4182]
Random effects model 0.3965 [0.2766; 0.5231]
Heterogeneity: I^2 = 79.4%, τ^2 = 0.0067, p = 0.0276
subgroup = other solid tumor
Chen, B. 2024−NSCLC 14 69 0.2029 [0.1165; 0.3174]
Gao, X. 2023−solid tumor 37 83 0.4458 [0.3370; 0.5588]
Gao, X. 2023−ESCC 13 22 0.5909 [0.3635; 0.7929]
Gao, X. 2023−HCC 11 24 0.4583 [0.2555; 0.6718]
Frentzas, S. 2023−solid tumor 53 119 0.4454 [0.3543; 0.5393]
Common effect model 317 0.3990 [0.3446; 0.4545]
Random effects model 0.4123 [0.2884; 0.5418]
Heterogeneity: I^2 = 77.6%, τ^2 = 0.0156, p = 0.0013
Common effect model 1098 0.4247 [0.3952; 0.4545]
Random effects model 0.4493 [0.3715; 0.5284]
0.2 0.3 0.4 0.5 0.6 0.7
irAE Rate
Heterogeneity: I^2 = 79.9%, τ^2 = 0.0121, p < 0.0001
Test for subgroup differences (common effect): χ^2_2 = 15.59, df = 2 (p = 0.0004)
Test for subgroup differences (random effects): χ^2_2 = 3.49, df = 2 (p = 0.1745)
B
Study Events Total Proportion [95% CI]
subgroup = CC
Wu, X. 2024 22 226 0.0973 [0.0625; 0.1441]
Lou, H. 2024 9 45 0.2000 [0.1026; 0.3486]
Gao, X. 2023−CC 15 111 0.1351 [0.0724; 0.2094]
Common effect model 382 0.1170 [0.0859; 0.1519]
Random effects model 0.1279 [0.0785; 0.1866]
Heterogeneity: I^2 = 48.3%, τ^2 = 0.0025, p = 0.1443
subgroup = G/GEJ
Shen, L. 2025 38 305 0.1246 [0.0867; 0.1646]
Gao, X. 2024 14 94 0.1489 [0.0824; 0.2363]
Common effect model 399 0.1294 [0.0978; 0.1645]
Random effects model 0.1294 [0.0978; 0.1645]
Heterogeneity: I^2 = 0%, τ^2 = 0, p = 0.5105
subgroup = other solid tumor
Chen, B. 2024−NSCLC 4 69 0.0580 [0.0170; 0.1427]
Gao, X. 2023−solid tumor 11 83 0.1325 [0.0693; 0.2256]
Gao, X. 2023−ESCC 4 22 0.1818 [0.0519; 0.4028]
Gao, X. 2023−HCC 3 24 0.1250 [0.0266; 0.3236]
Frentzas, S. 2023−solid tumor 8 119 0.0672 [0.0295; 0.1282]
Common effect model 317 0.0877 [0.0571; 0.1232]
Random effects model 0.0917 [0.0539; 0.1372]
Heterogeneity: I^2 = 28.7%, τ^2 = 0.0017, p = 0.2305
Common effect model 1098 0.1114 [0.0928; 0.1314]
Random effects model 0.1131 [0.0893; 0.1391]
0.05 0.1 0.15 0.2 0.25 0.3 0.35 0.4
irAE Grade>=3 Rate
Heterogeneity: I^2 = 25.7%, τ^2 = 0.0010, p = 0.2070
Test for subgroup differences (common effect): χ^2_2 = 2.20, df = 2 (p = 0.3326)
Test for subgroup differences (random effects): χ^2_2 = 1.40, df = 2 (p = 0.4959)
C
Study Events Total Proportion [95% CI]
subgroup = G/GEJ
Shen, L. 2025 23 305 0.0754 [0.0484; 0.1110]
Gao, X. 2024 2 94 0.0213 [0.0026; 0.0748]
Common effect model 399 0.0595 [0.0378; 0.0855]
Random effects model 0.0485 [0.0098; 0.1113]
Heterogeneity: I^2 = 75.4%, τ^2 = 0.0053, p = 0.0438
subgroup = other solid tumor
Chen, B. 2024−NSCLC 1 69 0.0145 [0.0004; 0.0781]
Common effect model 468 0.0509 [0.0320; 0.0735]
Random effects model 0.0385 [0.0102; 0.0810]
0 0.02 0.04 0.06 0.08 0.1
irAE (Discontinuation) Rate
Heterogeneity: I^2 = 70.7%, τ^2 = 0.0039, p = 0.0330
Test for subgroup differences (common effect): χ^2_1 = 2.76, df = 1 (p = 0.0967)
Test for subgroup differences (random effects): χ^2_1 = 1.01, df = 1 (p = 0.3149)
D
Study Events Total Proportion [95% CI]
subgroup = CC
Gao, X. 2023−CC 0 111 0.0000 [0.0000; 0.0320]
subgroup = G/GEJ
Gao, X. 2024 0 94 0.0000 [0.0000; 0.0369]
subgroup = other solid tumor
Chen, B. 2024−NSCLC 0 69 0.0000 [0.0001; 0.0531]
Gao, X. 2023−solid tumor 0 83 0.0000 [0.0002; 0.0448]
Gao, X. 2023−ESCC 0 22 0.0000 [0.0000; 0.1544]
Gao, X. 2023−HCC 0 24 0.0000 [0.0000; 0.1425]
Common effect model 198 0.0000 [0.0000; 0.0076]
Random effects model 0.0000 [0.0000; 0.0076]
Heterogeneity: I^2 = 0%, τ^2 = 0, p = 0.9590
Common effect model 403 0.0000 [0.0000; 0.0032]
Random effects model 0.0000 [0.0000; 0.0032]
0 0.02 0.06 0.1 0.12
irAE (Death) Rate
Heterogeneity: I^2 = 0.0%, τ^2 = 0, p = 0.9939
Test for subgroup differences (common effect): χ^2_2 = 0.14, df = 2 (p = 0.9306)
Test for subgroup differences (random effects): χ^2_2 = 0.14, df = 2 (p = 0.9306)

Figure S1. Incidence of immune-related adverse events (irAEs) organized by cancer types. (A) all-grade irAEs; (B) grade ≥3 irAEs; (C) irAEs leading to treatment discontinuation; (D) irAEs associated with mortality.

Abbreviations: CC: cervical cancer; G/GEJ: gastric or gastroesophageal junction; NSCLC: non-small cell lung cancer; ESCC: esophageal squamous cell carcinoma; HCC: hepatic cell carcinoma.
