# Supplementary material for: The Safety of Cadonilimab: A Systematic Review and Single‐Arm Meta‐Analysis
Source: Cancer Med. 2025 Sep 3;14(17):e71210. doi: 10.1002/cam4.71210 (PMC12405967; doi:10.1002/cam4.71210)
Supplement: Supplementary file 2 — Figure S2: Incidence of immune‐related adverse events (irAEs) organized by dosing groups. [file CAM4-14-e71210-s001.pdf]

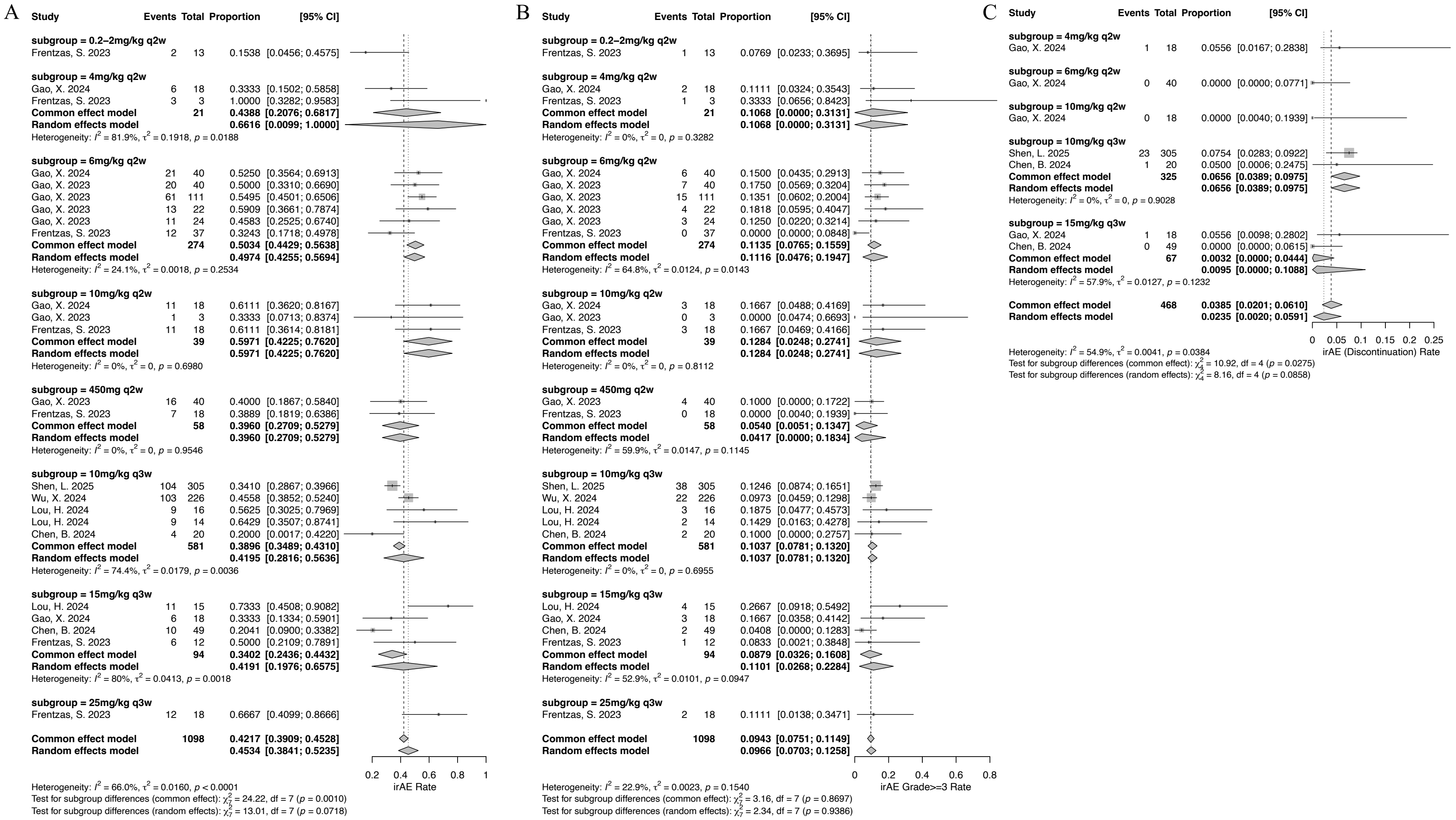

Figure S2. Incidence of immune-related adverse events (irAEs) organized by dosing groups. (A) all-grade irAEs; (B) grade ≥3 irAEs; (C) irAEs leading to treatment discontinuation.

Abbreviations: q2w: every 2 weeks; q3w: every 3 weeks
