# Supplementary material for: The Safety of Cadonilimab: A Systematic Review and Single‐Arm Meta‐Analysis
Source: Cancer Med. 2025 Sep 3;14(17):e71210. doi: 10.1002/cam4.71210 (PMC12405967; doi:10.1002/cam4.71210)
Supplement: Supplementary file 3 — Figure S3: Sensitive analysis of immune‐related adverse events (irAEs). [file CAM4-14-e71210-s003.pdf]

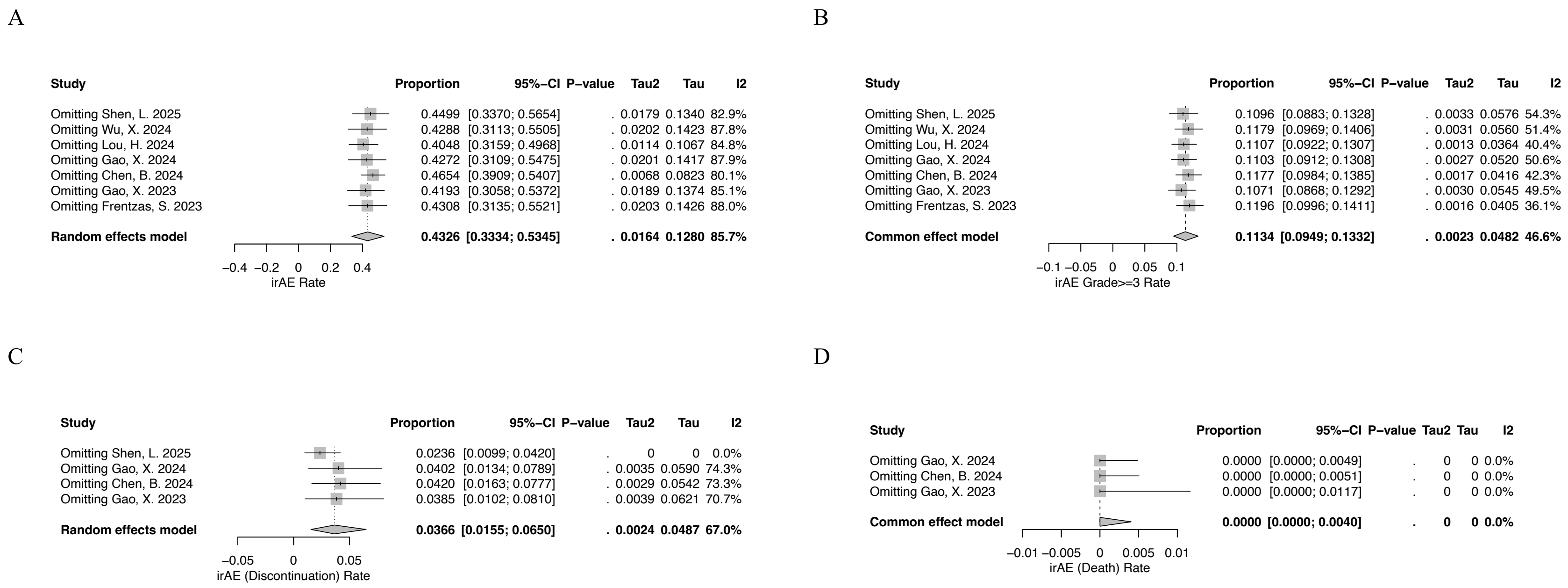

Figure S3. Sensitive analysis of immune-related adverse events (irAEs). (A) all-grade irAEs; (B) grade ≥3 irAEs; (C) irAEs leading to treatment discontinuation.
