# Supplementary material for: The Safety of Cadonilimab: A Systematic Review and Single‐Arm Meta‐Analysis
Source: Cancer Med. 2025 Sep 3;14(17):e71210. doi: 10.1002/cam4.71210 (PMC12405967; doi:10.1002/cam4.71210)
Supplement: Supplementary file 4 — Figure S4: Sensitive analysis of specific immune‐related adverse events (irAEs). [file CAM4-14-e71210-s007.pdf]

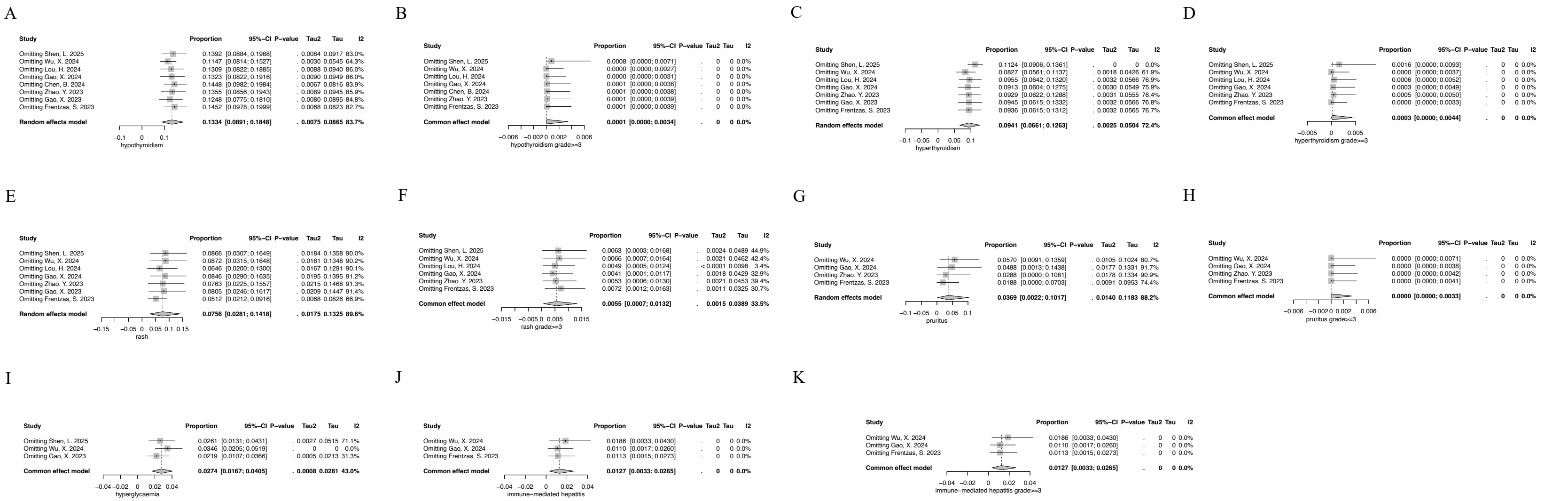

Figure S4. Sensitive analysis of specific immune-related adverse events (irAEs). (A) hypothyroidism; (B) hypothyroidism grade ≥3; (C) hyperthyroidism; (D) hyperthyroidism grade ≥3; (E) rash; (F) rash grade ≥3; (G) pruritus; (H) pruritus grade ≥3; (I) hyperglycaemia; (J) immune-mediated hepatitis; (K) immune-mediated hepatitis grade ≥3.
