# Supplementary material for: The Safety of Cadonilimab: A Systematic Review and Single‐Arm Meta‐Analysis
Source: Cancer Med. 2025 Sep 3;14(17):e71210. doi: 10.1002/cam4.71210 (PMC12405967; doi:10.1002/cam4.71210)
Supplement: Supplementary file 5 — Table S1: Risk of bias in randomized trials. [file CAM4-14-e71210-s006.doc]

Table S1. Risk of bias in randomized trials 1

| Study | Randomization process | Deviations from intended interventions | Missing outcome data | Measurement of the outcome | Selection of the reported result | Overall Bias |
| --- | --- | --- | --- | --- | --- | --- |
| Shen, L. 2025 | Low | Low | Low | Low | Low | Low |
| Wu, X. 2024 | Low | Some concerns | Low | Low | Low | Some concerns |
