# Supplementary material for: The Safety of Cadonilimab: A Systematic Review and Single‐Arm Meta‐Analysis
Source: Cancer Med. 2025 Sep 3;14(17):e71210. doi: 10.1002/cam4.71210 (PMC12405967; doi:10.1002/cam4.71210)
Supplement: Supplementary file 6 — Table S2: The revised and validated version of MINORS. [file CAM4-14-e71210-s004.doc]

Table S2. The revised and validated version of MINORS

| Methodological items for nonrandomized studies | Lou, H.  2024 | Long, B.  2024 | Gao, X.  2024 | Chen, Q. 2024 | Chen, B.  2024 | Zhao, Y.  2023 | Qiao, Q.  2023 | Gao, X.  2023 | Frentzas, S. 2023 |
| --- | --- | --- | --- | --- | --- | --- | --- | --- | --- |
| 1 | 2 | 2 | 2 | 2 | 2 | 2 | 2 | 2 | 2 |
| 2 | 2 | 2 | 2 | 2 | 2 | 2 | 2 | 2 | 2 |
| 3 | 2 | 2 | 2 | 2 | 2 | 2 | 2 | 2 | 2 |
| 4 | 2 | 2 | 2 | 2 | 2 | 2 | 2 | 2 | 2 |
| 5 | 0 | 0 | 0 | 0 | 0 | 0 | 0 | 0 | 0 |
| 6 | 2 | 2 | 2 | 2 | 2 | 2 | 2 | 2 | 2 |
| 7 | 2 | 2 | 2 | 2 | 2 | 2 | 2 | 2 | 2 |
| 8 | 0 | 2 | 2 | 0 | 2 | 2 | 2 | 2 | 2 |
| 9 | 0 | 0 | 0 | 0 | 0 | 0 | 0 | 0 | 0 |
| 10 | 0 | 0 | 0 | 0 | 0 | 0 | 0 | 0 | 0 |
| 11 | 2 | 2 | 2 | 2 | 2 | 2 | 2 | 2 | 2 |
| 12 | 2 | 2 | 2 | 1 | 2 | 1 | 1 | 2 | 2 |
| Total | 16 | 18 | 18 | 15 | 18 | 17 | 17 | 18 | 18 |

Abbreviations: MINORS: Methodological index for nonrandomized studies.

Methodological items for nonrandomized studies:

1. A clearly stated aim: the question addressed should be precise and relevant considering the available literature.

2. Inclusion of consecutive patients: all patients potentially ﬁt for inclusion (satisfying the criteria for inclusion) were included in the study during the study period (no exclusion or details about the reasons for exclusion).

3. Prospective collection of data: data were collected according to a protocol established before the beginning of the study.

4. Endpoints appropriate to the aim of the study: unambiguous explanation of the criteria used to evaluate the main outcome, which should be in accordance with the question addressed by the study. In addition, the endpoints should be assessed on an intention-to-treat basis.

5. Unbiased assessment of the study endpoint: a blind evaluation of objective endpoints and a double-blind evaluation of subjective endpoints. Otherwise, the reasons for not blinding should be stated.

6. Follow-up period appropriate to the aim of the study: the follow-up should be sufficiently long to allow the assessment of the main endpoint and possible adverse events.

7. Loss to follow-up of less than 5%: All patients should be included in the follow-up. Otherwise, the proportion lost to follow-up should not exceed the proportion experiencing the major endpoint.

8. Prospective calculation of the study size: information on the size of the detectable difference of interest with a calculation of the 95% confidence interval according to the expected incidence of the outcome event, and information about the level of statistical significance and estimates of power when comparing the outcomes. Additional criteria in the case of a comparative study.

9. An adequate control group: having a gold standard diagnostic test or therapeutic intervention recognized as the optimal intervention according to the available published data.

10. Contemporary groups: control and study groups should be managed during the same period (no historical comparison).

11. Baseline equivalence of groups: the groups should be similar regarding criteria other than the study endpoints under study. Absence of confounding factors that could cause bias in the interpretation of the results.

12. Adequate statistical analyses: whether the statistics were in accordance with the type of study with the calculation of confidence intervals or relative risk.

The items were scored as 0 (not reported), 1 (reported but inadequate), or 2 (reported and adequate). The global ideal score was 16 for noncomparative studies and 24 for comparative studies.
