# Supplementary material for: The Safety of Cadonilimab: A Systematic Review and Single‐Arm Meta‐Analysis
Source: Cancer Med. 2025 Sep 3;14(17):e71210. doi: 10.1002/cam4.71210 (PMC12405967; doi:10.1002/cam4.71210)
Supplement: Supplementary file 7 — Table S3: Summary of irAEs. [file CAM4-14-e71210-s002.docx]

Table S3 Summary of irAEs

| study_id | Shen, L. 2025 | Wu, X. 2024 | Lou, H. 2024 | Long, B. 2024 | Gao, X. 2024 | Chen, Q. 2024 | Chen, B. 2024 | Zhao. Y. 2023 | Qiao, Q. 2023 | Gao, X. 2023 | Frentzas, S. 2023 |
| --- | --- | --- | --- | --- | --- | --- | --- | --- | --- | --- | --- |
| hypothyroidism, n | 31 | 61 | 7 | NR | 13 | NR | 4 | 6 | NR | 45 | 8 |
| hypothyroidism ≥3, n | 0 | 1 | 1 | NR | 0 | NR | 0 | 0 | NR | NR | 0 |
| hyperglycaemia, n | 9 | 3 | NR | NR | NR | NR | NR | NR | NR | 10 | NR |
| hyperglycaemia ≥3, n | 4 | 1 | NR | NR | NR | NR | NR | NR | NR | NR | NR |
| hyperthyroidism, n | 12 | 33 | 4 | NR | 11 | NR | NR | 6 | NR | 23 | 12 |
| hyperthyroidism ≥3, n | 0 | 1 | 0 | NR | 0 | NR | NR | 0 | NR | NR | 1 |
| rash, n | 9 | 6 | 8 | NR | 3 | NR | NR | 4 | NR | 13 | 30 |
| rash ≥3, n | 2 | 1 | 2 | NR | 2 | NR | NR | 1 | NR | NR | 0 |
| thyroiditis, n | NR | 8 | NR | NR | NR | NR | NR | 1 | NR | NR | NR |
| thyroiditis ≥3, n | NR | 0 | NR | NR | NR | NR | NR | 0 | NR | NR | NR |
| immune-mediated thyroiditis, n | NR | 5 | NR | NR | NR | NR | NR | NR | NR | NR | NR |
| immune-mediated thyroiditis ≥3, n | NR | 1 | NR | NR | NR | NR | NR | NR | NR | NR | NR |
| TSH decreased, n | NR | NR | NR | NR | NR | NR | NR | 2 | NR | 11 | NR |
| TSH decreased ≥3, n | NR | NR | NR | NR | NR | NR | NR | 0 | NR | NR | NR |
| adrenal insufficiency, n | NR | 5 | NR | NR | NR | NR | NR | NR | NR | NR | 1 |
| adrenal insufficiency ≥3, n | NR | 0 | NR | NR | NR | NR | NR | NR | NR | NR | 0 |
| hypopituitarism, n | NR | 3 | NR | NR | NR | NR | 3 | NR | NR | NR | NR |
| hypopituitarism ≥3, n | NR | 2 | NR | NR | NR | NR | 1 | NR | NR | NR | NR |
| drug eruption, n | NR | 3 | NR | NR | NR | NR | NR | NR | NR | NR | NR |
| drug eruption ≥3, n | NR | 1 | NR | NR | NR | NR | NR | NR | NR | NR | NR |
| blood TSH increased, n | NR | 3 | NR | NR | NR | NR | NR | NR | NR | 17 | NR |
| blood TSH increased ≥3, n | NR | 0 | NR | NR | NR | NR | NR | NR | NR | NR | NR |
| secondary hyperthyroidism, n | NR | 3 | NR | NR | NR | NR | NR | NR | NR | NR | NR |
| secondary hyperthyroidism ≥3, n | NR | 0 | NR | NR | NR | NR | NR | NR | NR | NR | NR |
| immune-mediated hepatitis, n | NR | 2 | NR | NR | 2 | NR | NR | NR | NR | NR | 2 |
| immune-mediated hepatitis ≥3, n | NR | 2 | NR | NR | 2 | NR | NR | NR | NR | NR | 2 |
| Autoimmune hepatitis, n | NR | NR | NR | NR | NR | NR | 1 | NR | NR | NR | NR |
| Autoimmune hepatitis ≥3, n | NR | NR | NR | NR | NR | NR | 1 | NR | NR | NR | NR |
| diabetic ketoacidosis, n | NR | 2 | NR | NR | NR | NR | NR | NR | NR | NR | NR |
| diabetic ketoacidosis ≥3, n | NR | 2 | NR | NR | NR | NR | NR | NR | NR | NR | NR |
| ketoacidosis, n | NR | NR | NR | NR | NR | NR | 1 | NR | NR | NR | NR |
| ketoacidosis ≥3, n | NR | NR | NR | NR | NR | NR | 1 | NR | NR | NR | NR |
| primary hypothyroidism, n | NR | 2 | NR | NR | NR | NR | NR | NR | NR | NR | NR |
| primary hypothyroidism ≥3, n | NR | 0 | NR | NR | NR | NR | NR | NR | NR | NR | NR |
| hypophysitis, n | NR | 2 | NR | NR | NR | NR | NR | NR | NR | NR | NR |
| hypophysitis ≥3, n | NR | 1 | NR | NR | NR | NR | NR | NR | NR | NR | NR |
| lymphocytic hypophysitis, n | NR | NR | NR | NR | NR | NR | 2 | NR | NR | NR | 1 |
| lymphocytic hypophysitis ≥3, n | NR | NR | NR | NR | NR | NR | 0 | NR | NR | NR | 0 |
| hepatic function abnormal, n | NR | 2 | 3 | NR | NR | NR | NR | NR | NR | NR | NR |
| jepatic function abnormal ≥3, n | NR | 1 | 0 | NR | NR | NR | NR | NR | NR | NR | NR |
| myositis, n | NR | 2 | NR | NR | NR | NR | NR | NR | NR | NR | NR |
| myositis ≥3, n | NR | 0 | NR | NR | NR | NR | NR | NR | NR | NR | NR |
| increased AST, n | NR | 1 | NR | NR | NR | NR | NR | NR | NR | NR | NR |
| increased AST ≥3, n | NR | 1 | 3 | NR | NR | NR | NR | NR | NR | NR | NR |
| thyrotoxic crisis, n | NR | 1 | 0 | NR | NR | NR | NR | NR | NR | NR | NR |
| thyrotoxic crisis ≥3, n | NR | 1 | NR | NR | NR | NR | NR | NR | NR | NR | NR |
| immune-mediated hypophysitis, n | NR | 1 | NR | NR | NR | NR | NR | NR | NR | NR | NR |
| immune-mediated hypophysitis ≥3, n | NR | 1 | NR | NR | NR | NR | NR | NR | NR | NR | NR |
| diabetes mellitus, n | NR | 1 | NR | NR | NR | NR | NR | NR | NR | NR | NR |
| diabetes mellitus ≥3, n | NR | 1 | NR | NR | NR | NR | NR | NR | NR | NR | NR |
| type 1 diabetes mellitus, n | NR | 1 | NR | NR | NR | NR | NR | NR | NR | NR | 2 |
| type 1 diabetes mellitus ≥3, n | NR | 1 | NR | NR | NR | NR | NR | NR | NR | NR | 1 |
| dermatitis, n | NR | 1 | NR | NR | NR | NR | NR | NR | NR | NR | NR |
| dermatitis ≥3, n | NR | 1 | NR | NR | NR | NR | NR | NR | NR | NR | NR |
| increased GGT, n | NR | 1 | NR | NR | NR | NR | NR | NR | NR | NR | NR |
| increased GGT ≥3, n | NR | 1 | NR | NR | NR | NR | NR | NR | NR | NR | NR |
| arthritis, n | NR | 1 | NR | NR | NR | NR | NR | NR | NR | NR | 3 |
| arthritis ≥3, n | NR | 1 | NR | NR | NR | NR | NR | NR | NR | NR | 0 |
| immune-mediated arthritis, n | NR | NR | NR | NR | NR | NR | 1 | NR | NR | NR | NR |
| immune-mediated arthritis ≥3, n | NR | NR | NR | NR | NR | NR | 1 | NR | NR | NR | NR |
| immune-mediated myositis, n | NR | 1 | NR | NR | NR | NR | 1 | NR | NR | NR | NR |
| immune-mediated myositis ≥3, n | NR | 1 | NR | NR | NR | NR | 0 | NR | NR | NR | NR |
| pneumonitis, n | NR | 1 | NR | NR | 1 | NR | NR | NR | NR | NR | NR |
| pneumonitis ≥3, n | NR | 1 | NR | NR | 0 | NR | NR | NR | NR | NR | NR |
| immune-mediated Pneumonitis, n | NR | NR | NR | NR | NR | NR | 1 | NR | NR | NR | NR |
| immune-mediated Pneumonitis ≥3, n | NR | NR | NR | NR | NR | NR | 0 | NR | NR | NR | NR |
| uveitis, n | NR | 1 | NR | NR | NR | NR | NR | NR | NR | NR | NR |
| uveitis ≥3, n | NR | 1 | NR | NR | NR | NR | NR | NR | NR | NR | NR |
| immune-mediated myocarditis, n | NR | 1 | NR | NR | 1 | NR | NR | NR | NR | NR | NR |
| immune-mediated myocarditis ≥3, n | NR | 1 | NR | NR | 1 | NR | NR | NR | NR | NR | NR |
| erythema multiforme, n | NR | 1 | NR | NR | NR | NR | NR | NR | NR | NR | NR |
| erythema multiforme ≥3, n | NR | 0 | NR | NR | NR | NR | NR | NR | NR | NR | NR |
| pruritus, n | NR | 1 | NR | NR | 1 | NR | NR | 4 | NR | NR | 13 |
| pruritus ≥3, n | NR | 0 | NR | NR | 0 | NR | NR | 0 | NR | NR | 0 |
| stevens-Johnson syndrome, n | NR | 1 | NR | NR | NR | NR | NR | NR | NR | NR | NR |
| stevens-Johnson syndrome ≥3, n | NR | 0 | NR | NR | NR | NR | NR | NR | NR | NR | NR |
| thyroxine free increased, n | NR | 1 | NR | NR | NR | NR | NR | NR | NR | NR | NR |
| thyroxine free increased ≥3, n | NR | 0 | NR | NR | NR | NR | NR | NR | NR | NR | NR |
| autoimmune thyroiditis, n | NR | 1 | NR | NR | NR | NR | NR | NR | NR | NR | NR |
| autoimmune thyroiditis ≥3, n | NR | 0 | NR | NR | NR | NR | NR | NR | NR | NR | NR |
| hashitoxicosis, n | NR | 1 | NR | NR | NR | NR | NR | NR | NR | NR | NR |
| hashitoxicosis ≥3, n | NR | 0 | NR | NR | NR | NR | NR | NR | NR | NR | NR |
| thyroid disorder, n | NR | 1 | NR | NR | NR | NR | NR | NR | NR | NR | NR |
| thyroid disorder ≥3, n | NR | 0 | NR | NR | NR | NR | NR | NR | NR | NR | NR |
| glucocorticoid deficiency, n | NR | 1 | NR | NR | NR | NR | NR | NR | NR | NR | NR |
| glucocorticoid deficiency ≥3, n | NR | 0 | NR | NR | NR | NR | NR | NR | NR | NR | NR |
| secondary adrenocortical insufficiency, n | NR | 1 | NR | NR | NR | NR | NR | NR | NR | NR | NR |
| secondary adrenocortical insufficiency ≥3, n | NR | 0 | NR | NR | NR | NR | NR | NR | NR | NR | NR |
| cortisol abnormal, n | NR | NR | NR | NR | NR | NR | 1 | NR | NR | NR | NR |
| cortisol abnormal ≥3, n | NR | NR | NR | NR | NR | NR | 0 | NR | NR | NR | NR |
| drug-induced liver injury, n | NR | 1 | NR | NR | NR | NR | NR | NR | NR | NR | NR |
| drug-induced liver injury ≥3, n | NR | 0 | NR | NR | NR | NR | NR | NR | NR | NR | NR |
| myocarditis, n | NR | 1 | NR | NR | NR | NR | NR | NR | NR | NR | NR |
| myocarditis ≥3, n | NR | 0 | NR | NR | NR | NR | NR | NR | NR | NR | NR |
| myocardial injury, n | NR | 1 | NR | NR | NR | NR | NR | NR | NR | NR | NR |
| myocardial injury ≥3, n | NR | 0 | NR | NR | NR | NR | NR | NR | NR | NR | NR |
| sjogren's syndrome, n | NR | 1 | NR | NR | NR | NR | NR | NR | NR | NR | NR |
| sjogren's syndrome ≥3, n | NR | 0 | NR | NR | NR | NR | NR | NR | NR | NR | NR |
| increased ALT, n | NR | 0 | 5 | NR | NR | NR | NR | NR | NR | NR | NR |
| Increased ALT ≥3, n | NR | 0 | 0 | NR | NR | NR | NR | NR | NR | NR | NR |
| autoimmune thyroid disorder, n | NR | 0 | NR | NR | NR | NR | NR | NR | NR | NR | NR |
| autoimmune thyroid disorder ≥3, n | NR | 0 | NR | NR | NR | NR | NR | NR | NR | NR | NR |
| immune-mediated lung disease, n | NR | 0 | NR | NR | NR | NR | NR | NR | NR | NR | NR |
| immune-mediated lung disease ≥3, n | NR | 0 | NR | NR | NR | NR | NR | NR | NR | NR | NR |
| decrease PLT, n | NR | 0 | NR | NR | NR | NR | NR | NR | NR | NR | NR |
| decrease PLT ≥3, n | NR | 0 | NR | NR | NR | NR | NR | NR | NR | NR | NR |
| infusion-related reaction, n | NR | 0 | 4 | NR | NR | NR | NR | NR | NR | NR | NR |
| infusion-related reaction ≥3, n | NR | 0 | 0 | NR | NR | NR | NR | NR | NR | NR | NR |
| amylase increased, n | NR | NR | 2 | NR | NR | NR | NR | NR | NR | NR | NR |
| amylase increased ≥3, n | NR | NR | 0 | NR | NR | NR | NR | NR | NR | NR | NR |
| colitis, n | NR | NR | NR | NR | 2 | NR | NR | NR | NR | NR | NR |
| colitis ≥3, n | NR | NR | NR | NR | 1 | NR | NR | NR | NR | NR | NR |
| autoimmune colitis, n | NR | NR | NR | NR | 1 | NR | NR | NR | NR | NR | NR |
| autoimmune colitis ≥3, n | NR | NR | NR | NR | 1 | NR | NR | NR | NR | NR | NR |
| ILD, n | NR | NR | NR | NR | 1 | NR | NR | NR | NR | NR | NR |
| ILD ≥3, n | NR | NR | NR | NR | 0 | NR | NR | NR | NR | NR | NR |
| osteoarthritis, n | NR | NR | NR | NR | NR | NR | 1 | NR | NR | NR | NR |
| osteoarthritis ≥3, n | NR | NR | NR | NR | NR | NR | 0 | NR | NR | NR | NR |
| arthralgia, n | NR | NR | NR | NR | NR | NR | NR | NR | NR | NR | 7 |
| arthralgia ≥3, n | NR | NR | NR | NR | NR | NR | NR | NR | NR | NR | 0 |
| dry mouth, n | NR | NR | NR | NR | NR | NR | NR | NR | NR | NR | 2 |
| dry mouth ≥3, n | NR | NR | NR | NR | NR | NR | NR | NR | NR | NR | 0 |
| tendonitis, n | NR | NR | NR | NR | NR | NR | NR | NR | NR | NR | 1 |
| dry mouth ≥3, n | NR | NR | NR | NR | NR | NR | NR | NR | NR | NR | 0 |
| transaminase increased, n | NR | NR | NR | NR | NR | NR | NR | NR | NR | NR | 5 |
| transaminase increased ≥3, n | NR | NR | NR | NR | NR | NR | NR | NR | NR | NR | 1 |
| diarrhea, n | NR | NR | NR | NR | NR | NR | NR | NR | NR | NR | 4 |
| diarrhea≥3, n | NR | NR | NR | NR | NR | NR | NR | NR | NR | NR | 1 |
| immune-mediated enterocolitis, n | NR | NR | NR | NR | NR | NR | NR | NR | NR | NR | 1 |
| immune-mediated enterocolitis ≥3, n | NR | NR | NR | NR | NR | NR | NR | NR | NR | NR | 1 |
| polyneuropathy, n | NR | NR | NR | NR | NR | NR | NR | NR | NR | NR | 1 |
| polyneuropathy≥3, n | NR | NR | NR | NR | NR | NR | NR | NR | NR | NR | 1 |
| nephritis, n | NR | NR | NR | NR | NR | NR | NR | NR | NR | NR | 1 |
| nephritis≥3, n | NR | NR | NR | NR | NR | NR | NR | NR | NR | NR | 1 |
| iridocyclitis, n | NR | NR | NR | NR | NR | NR | NR | NR | NR | NR | 1 |
| iridocyclitis ≥3, n | NR | NR | NR | NR | NR | NR | NR | NR | NR | NR | 0 |

Abbreviations: irAEs: immune-related adverse events; TSH: thyroid-stimulating hormone; AST: aspartate transaminase; GGT: Gamma-glutamyl transferase; ALT: alanine transaminase; PLT: platelet; ILD: interstitial lung disease; NR: Not reported.
